# DNA Damage in Plant Herbarium Tissue

**DOI:** 10.1371/journal.pone.0028448

**Published:** 2011-12-05

**Authors:** Martijn Staats, Argelia Cuenca, James E. Richardson, Ria Vrielink-van Ginkel, Gitte Petersen, Ole Seberg, Freek T. Bakker

**Affiliations:** 1 Biosystematics Group, Wageningen University, Wageningen, The Netherlands; 2 Laboratory of Molecular Systematics, Natural History Museum of Denmark, University of Copenhagen, Copenhagen, Denmark; 3 Tropical Diversity Section, Royal Botanic Garden Edinburgh, Edinburgh, United Kingdom; 4 Laboratorio de Botánica y Sistemática, Universidad de Los Andes, Bogotá, Colombia; Institut de Biologia Evolutiva - Universitat Pompeu Fabra, Spain

## Abstract

Dried plant herbarium specimens are potentially a valuable source of DNA. Efforts to obtain genetic information from this source are often hindered by an inability to obtain amplifiable DNA as herbarium DNA is typically highly degraded. DNA post-mortem damage may not only reduce the number of amplifiable template molecules, but may also lead to the generation of erroneous sequence information. A qualitative and quantitative assessment of DNA post-mortem damage is essential to determine the accuracy of molecular data from herbarium specimens. In this study we present an assessment of DNA damage as miscoding lesions in herbarium specimens using 454-sequencing of amplicons derived from plastid, mitochondrial, and nuclear DNA. In addition, we assess DNA degradation as a result of strand breaks and other types of polymerase non-bypassable damage by quantitative real-time PCR. Comparing four pairs of fresh and herbarium specimens of the same individuals we quantitatively assess post-mortem DNA damage, directly after specimen preparation, as well as after long-term herbarium storage. After specimen preparation we estimate the proportion of gene copy numbers of plastid, mitochondrial, and nuclear DNA to be 2.4–3.8% of fresh control DNA and 1.0–1.3% after long-term herbarium storage, indicating that nearly all DNA damage occurs on specimen preparation. In addition, there is no evidence of preferential degradation of organelle versus nuclear genomes. Increased levels of C→T/G→A transitions were observed in old herbarium plastid DNA, representing 21.8% of observed miscoding lesions. We interpret this type of post-mortem DNA damage-derived modification to have arisen from the hydrolytic deamination of cytosine during long-term herbarium storage. Our results suggest that reliable sequence data can be obtained from herbarium specimens.

## Introduction

The world's approximately 3400 herbaria (http://sciweb.nybg.org/science2/IndexHerbariorum.asp) contain an immense number of plant specimens covering virtually all known species, making herbaria not only invaluable assets for understanding plant biodiversity [1], [2], but also largely underutilised genomic treasure troves. The expansion of next-generation sequencing (NGS) capabilities will potentially open up possibilities for cost-effective sequencing of genomes from type specimens and rare or extinct species stored in herbaria [3]. Although, DNA extraction results in irreparable damage to specimens, which conflicts with their historic and scientific importance, typically only a few milligrams of herbarium material need to be sampled. Nevertheless for small herbarium specimens (e.g. some Brassicaceae) or type specimens this can be too much, as the whole specimen basically has to be sacrificed. Therefore, considerable effort has been spent on optimizing DNA extraction protocols [4]–[6]. Furthermore, herbarium DNA is typically highly degraded into low molecular weight fragments [7]–[9]. Up until twenty years ago, herbarium specimen preparation techniques were not aimed at preserving DNA. Thus commonly used collection methods involved chemical treatments of specimens with formalin or ethanol, both of which severely affect DNA preservation in plants [7], [10], [11].

The occurrence of apuric sites, deaminated cytosine residues, and oxidized guanine residues are the main types of damage known from studies *in vivo* and on ancient DNA [12], [13]. In living cells, such sites can have lethal consequences and are efficiently repaired [14]. Herbarium specimen preparation, however, induces high levels of metabolic and cellular stress responses and ultimately cell death resulting in irreparably damaged DNA [15]. The post-mortem DNA damage inflicted during specimen preparation may be higher in organelles, as they are the major source of reactive oxygen species (ROS) known to inflict oxidative nucleotide damage [16], [17]. Once preserved, specimens in all major herbaria are normally (but not continuously) protected from the damaging effects of ultraviolet light and stored at moderate temperatures and at relatively low humidity, and often subjected to a two-yearly −20°C freezing cycle.

Damaged nucleotides in herbarium DNA may result in damage-specific nucleotide mis-incorporations (miscoding lesions) by DNA polymerase enzymes during amplification [18], [19]. In contrast to such polymerase-by-passable damage, strand-breaks and other DNA modifications block polymerases and thus prevent amplification. Qualitative and quantitative assessment of DNA post-mortem damage is therefore essential to determine the accuracy of DNA sequence data from herbarium specimens. The first aim of this study was to assess DNA damage as a result of polymerase non-bypassable damage using quantitative real-time PCR for multiple plastid, mitochondrial, and nuclear DNA regions. Secondly, levels of miscoding lesions in herbarium DNA were assessed using 454-sequencing of amplicons derived from each of the three genomic compartments. Using fresh and herbarium specimens of up to 114 years old, taken from the same individuals, allows a quantitative assessment of post-mortem DNA damage. Post-mortem damage was assessed, i) directly after herbarium specimen preparation by comparing results from fresh tissue and young herbarium specimens, and ii) after long-term herbarium storage by comparing results from young and old (>65 yrs.) herbarium specimens. Through statistical comparison, we investigated whether polymerase misincorporation errors alone explain the levels of miscoding lesions observed or whether they represent true damage-derived lesions in herbarium DNA. Finally, levels of DNA damage in the plastid, mitochondrial, and nuclear genomic compartments were compared in order to test for preferential DNA degradation among them.

## Results

### Degree of DNA fragmentation caused by herbarium preservation

The oldest herbarium material used was a 114 year old specimen of *Liriodendron tulipifera*. The other specimens included were *Ginkgo biloba* (107 years old) and *Laburnum anagyroides* (65 years old). The DNA extracted from these specimens was typically highly degraded and DNA fragment sizes were mostly below 1 kb (Figure S1). DNA extracts from young herbarium specimens (i.e., dated 8 July 2010) dried for 18 hours at 60°C did not contain high molecular weight DNA, and the average fragment size was below 10 kb. DNA extracts prior to filter cleanup were brownish, whereas cleanup yielded clear extracts with A_260_/A_280_ ratios between 1.7 and 2.0.

DNA yields from old and young herbarium specimens were not significantly different (*P* = 1.000; Table S6) and ranged between 33.91 and 103.40 ng DNA/mg tissue (Table S1). DNA yields from fresh tissue were on average 4.4–5.4 times higher, which may reflect differences in fresh versus herbarium DNA extraction efficiencies as low molecular weight DNA (DNA molecules <100 bp and nucleotides) is known to be less-efficiently recovered.

Any potential damage to herbarium DNA is likely to affect amplification efficiencies and calculated threshold cycle (Ct)-values. Depending on the kind and extent of damage this may lead to erroneous detection of gene copy numbers in herbarium DNA due to PCR jumping artefacts [28]. In this study, however, this effect was likely to be limited, because the qPCR amplicon sizes (80–140 bp) were always shorter than maximum fragments sizes (≤1 kb) in herbarium extracts. Furthermore, no amplification inhibition was observed in tests with serially diluted herbarium DNA samples (results not shown). Amplicon copy numbers for all plastid, mitochondrial, and nuclear gene regions in fresh and herbarium tissues are presented in Table S5. Mean gene copy numbers were measured by calculating the mean of plastid, mitochondial or nuclear amplicon numbers across genes per genomic compartment (Table S6).

**Figure 1:**
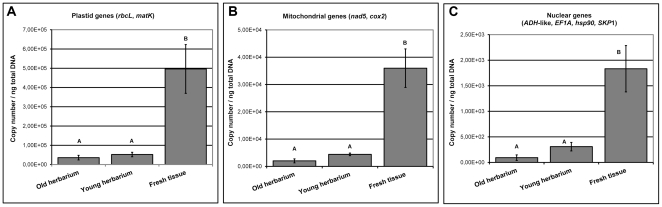
Copy numbers for herbarium specimens and fresh tissues for:(A) plastid genes (B) mitochondrial genes (C) nuclear genes. Values statistically different at 5% significance level in post-hoc tests are indicated by different letters (A or B).

**Figure 2:**
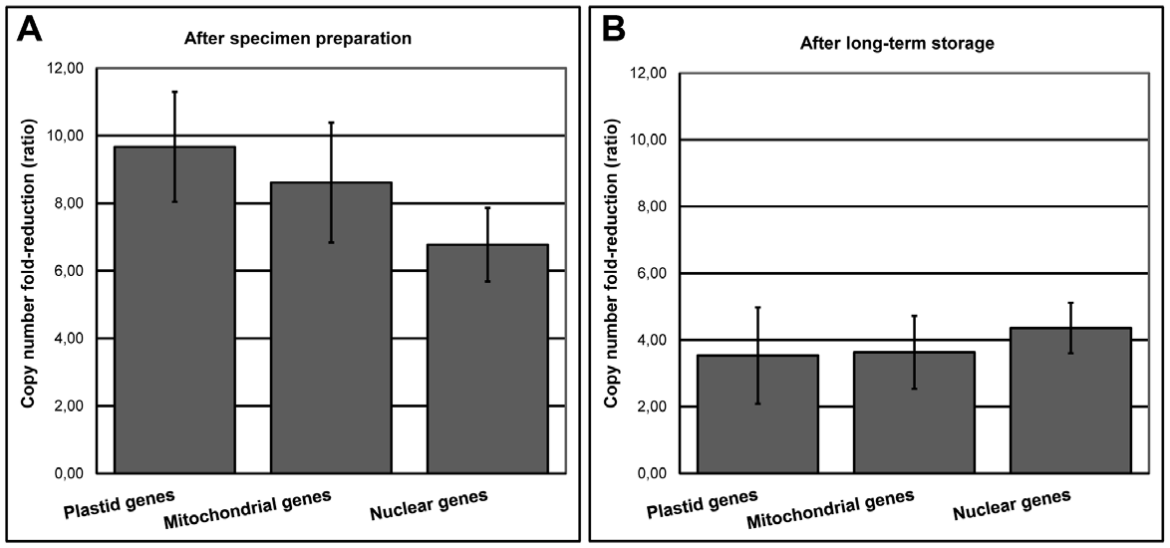
Copy number fold-reduction of plastid, mitochondrial and nuclear genes in herbarium specimens (A) after specimen preparation (fresh tissue/young herbarium ratio), and (B) after long-term herbarium storage (young/old herbarium ratio). Values were not statistically different at 5% significance level.

Fresh leaf tissue yielded significantly higher plastid, mitochondrial and nuclear gene copy numbers than young and old herbarium specimens (Figure 1A–C). As measured by the fresh tissue/young herbarium gene-copy ratio, plastid copy numbers after specimen preparation were on average 9.7-fold reduced (Figure 2A), which equates to a mean reduction of 444196 plastid DNA copies (per ng of total DNA) after specimen preparation (Table S6). Compared to young herbarium specimens, plastid copy numbers were on average 3.5-fold reduced following long-term storage (young/old herbarium ratio; Figure 2B), which equates to a mean reduction of 16322 plastid DNA copies after long-term storage. Likewise, mitochondrial DNA was reduced with 31589 copies after specimen preparation and 2373 copies after long-term storage, representing an mtDNA copy number reduction of 8.6 and 3.63-fold, respectively (Figure 2).

Due to the inability to produce optimal qPCR standard curves for some nuclear gene amplicons (results not shown), different sets of nuclear genes were assayed for the four species. Since the assayed loci were all predicted to be low-copy nuclear genes based on PLAZA 2.0 BLAST searches (Table S2), we believe it justifiable to compare copy numbers for different nuclear loci. Nuclear DNA was reduced with 1524 copies after specimen preparation and 215 copies after long-term storage, representing a nDNA copy number reduction of 6.8 and 4.3-fold, respectively (Figure 2).

Our results indicate that there is no difference in the degree of DNA fragmentation between plastid, mitochondrial, and nuclear genomes in herbarium specimens, as the reduction in nDNA copy numbers is not statistically different from those in plastid or mitochondrial DNA (Figure 2). Organelle DNA therefore does not appear to be preferentially degraded in herbarium tissue both directly following drying, or after long-term storage. Copy number reduction directly after drying was more pronounced than that following long-term storage. We estimate that, depending on the genomic compartment, 10.5–16.8% of the total DNA extracted from young herbarium specimens were amplifiable using *Taq* polymerase (Table 1). After correction for losses in DNA yield due to herbarium specimen preparation, however, we estimate the proportion of amplifiable copy numbers for plastid, mitochondrial, and nuclear DNA to be 2.4–3.8% of that of fresh controls and 1.0–1.3% after long-term storage.

**Table 1:** Mean DNA yield, mean gene copy numbers, and DNA yield loss-corrected copy numbers for plastid, mitochondrial and nuclear DNA relative to fresh control.

		% mean gene copy numbers^b^		
Sample type	% mean DNA yield^a^	Plastid	Mitochondrial	Nuclear
**Fresh tissue**	100	100	100	100
**Young herbarium**	22.6	10.5 (2.4)	12.2 (2.8)	16.8 (3.8)
**Old herbarium**	18.7	7.2 (1.3)	5.6 (1.0)	5.1 (1.0)

a Percentages were calculated relative to fresh controls, which were set at 100%. Mean DNA yields and mean gene copy numbers were taken from Table S6.

b Percentages of mean gene copy numbers corrected for DNA yield losses relative to fresh control (in brackets) are calculated as: (% mean DNA yield) × (% mean gene copy numbers).

### Elevated levels of C→T/G→A transitions in old herbarium plastid DNA

The average depth of coverage per amplicon was 4640×, and therefore the level of detection was sufficient to detect 1–5% variation of single base changes (Roche application note No. 5). The overall nucleotide misincorporation error rate recorded in fresh leaf tissue was ∼1.4×10^−3^ per nucleotide (44127 total substitutions/31933585 total nucleotides in fresh tissue; Table 2). This background substitution level in DNA extracted from fresh leaf tissue was assumed not only due to nucleotide misincorporations that arise from DNA polymerase errors, but also because of 454-sequencing errors and potential damage that may have arisen during DNA extraction.

**Table 2:** Chi-square (χ^2^) tests of independence (at *p*≥0.05 level) on number of nucleotide substitutions observed in fresh and herbarium DNA.

		Fresh tissue	Young herbarium			Old herbarium		
DNA type	Substitution type	Observed (%)^b^	Observed (%)	Observed, corrected^c^	÷^2^ contribution (*P*-value)	Observed (%)	Observed, corrected	÷^2^ contribution (*P*-value)
**Plastid**	**Total nucleotides** a	10746437	14193327	10746437		14101498	10746437	
	**Total substitutions**	11298	16047	12149.94		22527	17167.32	
	**(A→G/T→C)**	8504 (0.0791)	11634 (0.0817)	8808.65	10.91	15040 (0.1066)	11461.65	1028.66
	**(A→C/T→G)**	264 (0.0024)	427 (0.0030)	323.30	13.32	619 (0.0044)	471.72	163.45
	**(A→T/T→A)**	521 (0.0048)	585 (0.0041)	442.93	11.69	937 (0.0066)	714.07	71.54
	**(C→A/G→T)**	272 (0.0025)	386 (0.0027)	292.26	1.51	1021 (0.0072)	778.08	941.61
	**(C→G/G→C)**	49 (0.0046)	79 (0.0006)	59.82	2.39	98 (0.0007)	74.68	13.46
	**(C→T/G→A)**	1688 (0.0157)	2936 (0.0207)	2222.98	169.55	4812 (0.0341)	3667.12	2320.44
					209.37 (<0.001)			4539.16 (<0.001)
**Mitochondrial**	**Total nucleotides**	8566629	9769969	8566629		8738228	8566629	
	**Total substitutions**	14050	16174	14181.89		17277	16937.72	
	**(A→G/T→C)**	7580 (0.0885)	8828 (0.090)	7740.68	3.41	8113 (0.0928)	7953.68	18.42
	**(A→C/T→G)**	380 (0.0044)	408 (0.0042)	357.75	1.30	525 (0.0060)	514.69	47.74
	**(A→T/T→A)**	317 (0.0037)	403 (0.0041)	353.36	4.17	328 (0.0038)	321.56	0.07
	**(C→A/G→T)**	569 (0.0066)	634 (0.0065)	555.91	0.30	834 (0.0095)	817.62	108.63
	**(C→G/G→C)**	198 (0.0023)	247 (0.0025)	216.58	1.74	281 (0.0032)	275.48	30.32
	**(C→T/G→A)**	5006 (0.0584)	5654 (0.0579)	4957.61	0.47	7196 (0.0823)	7054.69	838.42
					11.39 (0.044)			1043.60 (<0.001)
**Nuclear**	**Total nucleotides**	12620519	13953891	12620519		8608206	12620519	
	**Total substitutions**	18779	20334	18390.97		21269	31182.55	
	**(A→G/T→C)**	13084 (0.1036)	13923 (0.0997)	12592.58	18.46	11726 (0.1362)	17191.53	1289.49
	**(A→C/T→G)**	547 (0.0043)	595 (0.0043)	538.14	0.14	826 (0.0096)	1211.00	806.03
	**(A→T/T→A)**	682 (0.0054)	702 (0.0050)	634.92	3.25	1380 (0.0160)	2023.22	2637.65
	**(C→A/G→T)**	473 (0.0037)	498 (0.0036)	450.41	1.08	681 (0.0079)	998.42	583.64
	**(C→G/G→C)**	243 (0.0019)	348 (0.0025)	314.75	21.18	417 (0.0048)	611.37	558.41
	**(C→T/G→A)**	3750 (0.0297)	4268 (0.0306)	3860.17	3.24	6239 (0.0725)	9147.02	7767.41
					47.35 (<0.001)			31642.64 (<0.001)

a Total numbers of adenine, thymine, cytosine and guanine in the dataset.

b Numbers of observed substitutions and their percentages of total nucleotides observed (in brackets).

c The numbers of observed substitutions were corrected for differences in total sequence data (nucleotides) between fresh and herbarium data.

Separate analyses were performed for plastid, mitochondrial, and nuclear DNA. Observed numbers in the fresh tissue data were used as expected numbers in χ^2^-tests.

Chi-square (χ^2^) tests of independence were used to compare the observed distributions of substitutions in herbarium DNA to the expected distributions in fresh leaves. Because the amplicons were generated by PCR, the actual strand of origin of potential miscoding lesions cannot be identified. Therefore, the data were summarized into six complementary pairs of nucleotide substitution. χ^2^ testing of the plastid data provided strong support that the quantitative distributions of substitutions summarized over the six substitution types in young herbarium specimens (χ^2^ = 209.37; df = 5; *P*<0.001) and old ones (χ^2^ = 4539.16; df = 5; *P*<0.001) were significantly different from those in fresh tissue (Table 2). Similarly high chi-square values were observed for mitochondrial and nuclear data (Table 2).

Analyses of variance (ANOVA) were performed for each of the six substitution types separately (Table S7). This enabled the identification of specific substitution types in herbarium specimens that were over-represented and thus may be attributed to DNA post-mortem damage. C→T/G→A transitions in plastid DNA from old herbarium specimens occur significantly more frequently than in plastid DNA from fresh and young herbarium (*F* = 37.42; *P*<0.001; Table S7). No increased levels of C→T/G→A transitions were detected in herbarium mitochondrial and nuclear DNA. Plastid C→T/G→A transitions constitute 21.4% (4812 out of 22527 substitutions) of the observed miscoding lesions in old herbarium plastid DNA (Table 2). Although, the overall percentage of total observed nucleotide sites at which the C→T/G→A transitions occurred is low (0.0341%), the C→T/G→A rate was approximately twice as high as in fresh tissue (0.0157%; Table 2). Based on our results therefore, the estimated C→T/G→A rate in herbarium plastid DNA during storage can be calculated to be 1.53*10^−6^ per nucleotide per year (Table 3). Furthermore, A→C/T→G transversions in nuclear DNA occurred significantly more frequently in old herbarium than in fresh material and young herbarium DNA (*F* = 10.74; *P* = 0.005; Table S7). However, they constitute only 3.9% (826 out of 21269 substitutions) of the total miscoding lesions, and appear to play little or no role in damage-derived miscoding lesions in herbarium DNA (Table 2).

**Table 3:** Estimated (C→T/G→A) rate in herbarium plastid DNA during herbarium storage.

	Number of substitutions per 10^6^ nucleotides			
Species	Young herbarium	Old herbarium	Old herbarium age (yrs.)	(C→T/G→A) per nucleotide per year ± SD
*Laburnum*	197.55	330.02	65	2.04*10^−6^
*Ginkgo*	229.09	384.02	107	1.45*10^−6^
*Liriodendron*	186.14	313.52	114	1.12*10^−6^
Average				1.53*10^−6^±4.66*10^−7^

## Discussion

Herbaria are major depositories for conserved plant material, and their combined collections provide an unparalleled record of the world's flora [1], [29]. It is not surprising, therefore, that herbarium specimens are a common source of DNA for studies on plant evolution. Despite this, little research has focused on post-mortem DNA damage in herbarium material and its influence on data quality. As far as we know, this is the first study to quantify the prevalent damage types in herbarium DNA following an experimental approach.

### Degree of DNA fragmentation caused by herbarium DNA preservation

Our results show that the most obvious form of post-mortem damage in herbarium DNA is double-stranded breaks. It is likely that the high temperatures (60–70°C) at which herbarium specimens are typically dried today causes cells to rupture quickly, concomitantly releasing nucleases, ROS, and other cellular enzymes. Such physiological conditions resemble necrosis, and this overwhelming cellular stress typically causes DNA to be degraded randomly into smaller fragments, appearing as a smear on agarose gels [30], [31]. Our experience is that DNA extracts from herbarium tissues rarely show oligo-nucleosomal DNA fragmentation [30], which is an indicator for programmed cell death (PCD). PCD is a highly coordinated death process, which is known to activate extra nucleases and ROS that damage DNA [30], [32]. Signs of DNA ‘laddering’ may indicate that the plant material was preserved too slowly and that the plant had experienced abiotic stresses for prolonged periods (hours, days) during preparation. Rapid desiccation of plant material has been shown to be the best way to preserve plant DNA [10], as it limits the PCD damage process.

Given that mtDNA and plastid DNA are close to major sites of ROS production and have no histones and no chromatine structure, one might expect higher levels of damage in organelle DNA during herbarium specimen preparation. However, no preferential degradation of mtDNA or plastid DNA was observed, and our results, therefore, indicate that DNA damage levels in each of the three genomic compartments are equal. The high temperatures at which herbarium specimens are typically dried most likely directly affects the degree to which DNA is preserved. Heating is known to greatly accelerate hydrolytic depurination, which in combination with the spontaneous cleavage of the phosphodiester backbone by ß-elimination will result in strand breaks [33], [34].

Our results indicate that up to 89.5% of DNA of young herbarium specimens may not be accessible to *Taq* polymerase (100% minus 10.5% mean plastid copy numbers; Table 1). The poor amplification success was not due to the presence of PCR inhibitors, as herbarium extracts were not found to delay amplification of an exogenous DNA control. Another possible explanation is that damage in herbarium DNA may be locus- or region-specific, and there is possibly large copy number heterogeneity in herbarium DNA samples. Gene copy numbers within each genomic compartment, however, were found to be of the same order of magnitude, rejecting a region-specific damage hypothesis. Admittedly, the representation of each genomic compartment by only two or three loci is a coarse representation of (the complexity of) organellar and nuclear genomes. A more representative sampling will be needed in order to exclude potential hotspots of degradation in herbarium DNA. A further consideration is that herbarium DNA was severely modified, not only by double stranded breaks, but also by inter-strand cross-links, abasic sites, or other structural modifications. These types of polymerase non-bypassable damage will also prevent DNA molecules from being amplified and sequenced. As an example, some studies on ancient DNA have concluded that DNA molecules were highly modified by blocking lesions [35], [36]. It will be of great value to assess the levels of abasic sites and other types of polymerase non-bypassable damage in herbarium DNA, as this will facilitate future DNA repair and rescuing strategies. Such strategies can help to improve the retrieval of accurate sequence information from herbarium specimens. For example, by pre-treating the herbarium DNA with enzymes involved in base excision repair, specific types of blocking lesions could be removed [37]. Furthermore, the use of engineered polymerases capable of extending beyond blocking lesions may allow for the amplification of highly damaged herbarium DNA molecules [38].

### Degree of DNA sequence modification in herbarium DNA

Increased levels of C→T/G→A transitions were observed in plastid DNA of old herbarium specimens as compared to young ones and fresh tissues. Therefore, polymerase and sequencing errors alone cannot account for all of the observed nucleotide substitutions (as we would expect these to occur without bias), which may be better explained by added misincorporations resulting from miscoding lesions present in old herbarium DNA molecules. The observed levels were, however, low (21.4% of miscoding lesions), and we found the C→T/G→A rate to be 1.53*10^−6^ per nucleotide per herbarium storage year. C→T/G→A (type 2) transitions are the most common form of miscoding lesions in ancient DNA [39], [40] and are ascribed to hydrolytic deamination of cytosine to uracil [18], [41]. Our results therefore indicate that even though herbarium specimens are protected from the greatest threats to their long-term maintenance, herbarium DNA remains susceptible to hydrolytic activity. It seems probable that moisture in the air may allow for some rehydration of DNA that will lead to hydrolytic DNA damage.

We have no comprehensive explanation for the fact that the observed increased levels of C→T/G→A transitions seem to be exclusive for herbarium plastid DNA. A possible explanation is that DNA damage is probably more easily detected in plastid genes, simply because of the far higher template copy numbers compared with mitochondrial and (low-copy) nuclear genes. To what extent GC-content could play a role is not clear: %GC is usually low in angiosperm plastid genomes [42], which would seem to contradict increased type 2 transition rate hypothesis.

Further evidence for DNA damage following long-term herbarium storage is obtained from the apparent higher levels of DNA fragmentation and associated gene copy number decrease in old (65–110 yrs.) herbarium versus young herbarium specimens (Table 1; Figure S1). It should be noted, however, that the levels of DNA damage in young and old herbarium specimens may not be directly compared. First, there is no record of the exact method that had been used to prepare the old herbarium specimens. For instance, the precise temperature at which they were dried is unknown. Moreover, the temperature of commonly used gas-ovens in the early 1900 s at the Leiden herbarium would have been difficult to control. Second, we did not account for potential seasonal and year-to-year fluctuations in DNA copy numbers in old herbarium specimens (although we collected our living specimens roughly at the same original date). Plastid DNA copy numbers are known to decline during the growing season, which is typical for maturing plant leaves [43]. Therefore, a precise quantification of DNA damage after long-term herbarium storage cannot be given. However, the present results clearly indicate that nearly all DNA damage occurred during specimen desiccation, and we estimate that only a small proportion of damage can be attributed to long-term storage (Table 1; Figure 1).

Our findings may not be directly applicable to herbarium material in general, as a large variety of desiccation methods have been used to preserve specimens. Only since the 1990 s was the routine established in herbaria to collect separate DNA samples in silica gel [10], along with the herbarium specimens. Common practice for field preparation, especially in the tropics, would have been the temporary fixation with methylated spirits (the ‘Schweinfurt method’) or 30% formaldehyde to prevent specimens from moulding. Unfortunately, use of these chemicals is known to have destructive effects on DNA [8]. In addition, there are numerous other techniques for field drying, including the use of kerosene stoves, 100-watt light-bulbs, and air-drying on a moving vehicle. Assessing implications of each method on DNA quality will not be straightforward, as the information on the exact drying method is typically not recorded. Moreover, factors that may influence the degree of DNA preservation are likely to be species-specific (e.g., contents of secondary metabolites, etc.) and relate to the physiological state of the plant when collected, and they are therefore difficult to predict [15].

### Implications

Our study confirms that herbaria are incredibly rich sources for reliable DNA sequence data. Various next-generation sequencing approaches can now be applied to severely fragmented DNA, and although more difficult it is still possible to generate DNA sequence data from them [44]. Nevertheless, our observation that polymerase-accessible DNA may be reduced by up to ∼90% could cause challenges in sample preparation using current NGS approaches, especially when limited amounts of herbarium material are available. Probably a more serious issue would be the degree of sequence modification. Our results indicate that C→T/G→A substitutions may theoretically cause an incorrect DNA sequence to be produced; however, we predict the sequence error rate to be negligible (∼0.03%) even if PCR products (i.e., the 750- bp *rbcL* barcode region sequence) had been sequenced from a single clone. However, the importance of these problems depends upon the type of investigation, e.g. random errors are like random noise unlikely to produce a phylogenetic signal. Indeed, so far there is no indication that, within the context of comparative studies including both fresh and old herbarium accessions, the latter share additional type 2 transitions.

The adoption of silica-gel drying of specimens generally yields higher quality DNA than most herbarium specimen preparation methods [10], and we recommend that all future collections stored for subsequent genetic analysis should continue to use this approach. However, our results confirm that herbarium DNA is a readily available resource that will be invaluable for future phylogenetic and genomic studies not least in view of the current biodiversity crisis. Other methods, e.g., proteomics or RNA studies, may require alternative conservation methods.

In a further study we will focus on elucidating the role of blocking lesions that may prevent sequencing of herbarium DNA. A study on this matter, using massive parallel sequencing of herbarium DNA, will be published elsewhere.

## Materials and Methods

### Specimen sampling and herbarium specimen preparation

Plant material (leaves) of living trees were sampled from the Leiden Botanical Garden and associated herbarium specimens (65–114 years old) of the same individuals were obtained from the collections of the National Herbarium of the Netherlands at Leiden (Table S1). All necessary permissions for the described plant and specimen sampling were obtained from the respective curators, i.e. Dr. Paul J.A. Keβler (Hortus Leiden) and Dr. Jan de Koning (Herbarium Leiden). By selecting fresh and herbarium material from the same individual we avoided possible intraspecific genetic variation (e.g., genotypic variation and variations in the amounts of cpDNA) although we acknowledge that some level of somatic variation may exist. *Ginkgo biloba* L. (Ginkgoaceae), *Laburnum anagyroides* Medik. (Fabaceae), *Liriodendron tulipifera* L. (Magnoliaceae), and *Lonicera maackii* (Rupr.) Herder (Caprifoliaceae) were sampled. Fresh leaves were sampled on 8^th^ July 2010 and stored at −80°C until DNA extraction. Herbarium specimens of fresh leaves from the same four species (i.e. dating 8 July 2010) were prepared by aluminium corrugate-drying [9] and are referred to hereafter as ‘young herbarium specimens’. In order to simulate common practice in herbaria, and hence to make our results representative for typical herbarium specimens, stacked samples were pressed and dried overnight in an electric oven (Binder, type IP20) at 60°C for 18 hours. As far as we could reconstruct, our old herbarium specimens had been preserved using a similar corrugate-drying method and at similar temperature.

### DNA extraction and purification

Total genomic DNA was extracted using a modified cetyl-trimethyl-ammonium-bromide (CTAB) method [20], probably the most commonly used plant DNA extraction method. Briefly, we used the following modifications: 50 mg of leaf material (with large veins removed) was weighed and placed into a 2 ml reaction tube containing five glass beads (Ø 3 mm). The reaction tube was submerged in liquid nitrogen, and the sample was then homogenized using beads (Retch, type MM2) for 30 seconds at 80 rpm. Liquid nitrogen freezing and homogenization were repeated until the leaf material had turned into a fine powder. One ml of CTAB buffer (2% CTAB, 2% PVP-40, 100 mM Tris-HCL, 1.4 M NaCl, 20 mM EDTA) and 12 µl β-mercaptoethanol were added with subsequent incubation for 60 min at 55°C. One ml of 24∶1 chloroform∶isoamylalcohol was then added, vortexed and centrifuged at 14.000 rpm for 4 min. The supernatant was removed, and the chloroform extraction was repeated. DNA was precipitated using 70% isopropanol at −20°C for 2 weeks, after which the DNA was pelleted at 14.000 rpm for 5 min. DNA was then re-suspended in TE buffer and treated with RNAse (Qiagen). DNA purification was performed using the Wizard DNA clean-up system (Promega Corp.) in combination with a vacuum manifold (Promega Corp.). The DNA was dissolved in 75 µl of pre-heated elution buffer (Qiagen). DNA extractions were visualized on 1% agarose gels (Figure S1), and the quantity was measured using a NanoDrop 1000 spectrophotometer (Thermo Scientific). DNA extractions were performed in duplicate or triplicate, and DNA yield per dry weight tissue was determined.

### Amplification and sequencing of plastid, mitochondrial and nuclear target genes

Multiple target regions were selected for primer design, of which Expressed Sequence Tag (EST) data and/or other (partial) sequence data was available on GenBank for the plant species of interest or for closely related species. Primers were designed using Primer3Plus [21] to amplify regions of the two plastid genes (*matK*, *rbcL*) from which regions have been selected as barcode markers for land plants [22], two mitochondrial genes (*coxII*, *nad5*), and six low-copy nuclear genes (*ADH*-like, *EF1A*, *H3*, *hsp90*, *RD19*-like, *SKP1*), as well as the 18S rDNA gene (Table S2). These (high-quality and full-length) reference sequences were used for the subsequent design of nested primers for use in real-time PCR assays and 454-sequencing (see below).

PCR amplification was carried out in a 25-µl reaction mixture that contained 10 to 50 ng of total DNA isolated from fresh plant material, 1× DreamTaq™ buffer (Fermentas), 20 µg BSA, 0.2 mM of each deoxynucleoside triphosphate (Promega), 10 µM of each primer (Biolegio), and 1.0 U of DreamTaq™ DNA polymerase (Fermentas). The following thermocycling pattern was used to amplify each gene fragment: 94°C for 5 min (1 cycle); 94°C for 30 s, 55°C for 30 s and 72°C for 60 s (35 cycles); and then 72°C for 8 min (1 cycle). PCR products were purified using the Qiaquick PCR purification kit (Qiagen) following manufacturer's instructions. In order to verify sequence identity, purified PCR products were sequenced in both directions on an ABI9600 sequencer (Greenomics).

For *H3* and *SKP1* of *Laburnum anagyroides*, *ADH*-like of *Liriodendron tulipifera*, and *SKP1* of *Lonicera maackii* PCR products were cloned because PCR yields were insufficient for direct sequencing or because non-specific amplification products were detected. Clones were generated in *E. coli* using pGEM®-T Easy Vectors (Promega). Sequences were submitted to EMBL under accession numbers FR869989–FR870021. Reference gene (sub-) families, cellular component ontology, and gene family size of target regions were identified using BLAST searches against the PLAZA 2.0 platform for plant comparative genomics database at http://bioinformatics.psb.ugent.be/plaza/ [23].

### Real-time quantitative PCR (qPCR)

DNA from fresh and herbarium samples was subjected to qPCR assays targeting two plastid genes (*matK*, *rbcL*), two mitochondrial genes (*coxII*, *nad5*), and three of four low-copy nuclear genes (*ADH*-like, *EF1A*, *hsp90*, *SKP1*). PCR primers for real-time PCR assays, designed to amplify fragments between 88 to 140 bp, are reported in Table S3. Real-time detection of PCR products was conducted with SYBR Green I with the MyiQ detection system (Bio-Rad) and was conducted in a total volume of 20 µl, containing 1× iQ SYBR Green Supermix (Bio-Rad), 5 µM of both primers, 20 µg BSA, and 5 to 10 ng of total DNA. The temperature profile was: 95°C for 5 min (1 cycle); 95°C for 15 s, 60°C for 20 s, and 72°C for 20 s (40 cycles), with a final extension step at 72°C for 5 min. After amplification, melting curve analysis was performed from 60°C to 95°C, with increments of 0.5°C per 10 seconds. Real-time PCR standards were prepared by cloning target amplicons and subsequent amplification with M13 forward and reverse primers. The cloned PCR fragments were then purified and the yield was quantified using a NanoDrop 1000 (Thermo Scientific). Samples were analysed in triplicate (replicates of same DNA), and serial-diluted samples and no-template controls were included. Gene copy numbers were quantified absolutely by quantifying them per nanogram of total DNA. Amplification efficiencies for optimized qPCR assays calculated from slopes of the standard curves are shown in Table S3: efficiencies were between 1.90 and 2.09, with *R^2^*-values, a measure for the quality of the regression fit, between 0.92 and 0.99. An exogenous control sequence from the wingless gene (*Wg*) of *Cymothoe* Hüber 1819 sp. (Lepidoptera, Insecta) was spiked to test for inhibition of qPCR assays by substances in the plant DNA extracts, but no inhibition was observed (data not shown). Gene copy numbers were considered to be duplicate (e.g., *rbcL* and *matK*, or *nad5* and *cox2*) or triplicate measurements (*ADH*-like, *EF1A*, and *hsp90* in *Liriodendron tulipifera*) in one-way ANOVA analyses, performed in PASW Statistics version 18.0.2 (SPSS Inc.). Copy number fold-reductions of plastid, mitochondrial, and nuclear genes directly after specimen preparation were expressed as ‘fresh tissue/young herbarium gene copy number ratio’, whereas those after long-term storage were expressed as ‘young herbarium/old herbarium gene copy number ratio’.

### 454-sequencing of amplicons

Fusion primers for unidirectional 454 sequencing of amplicons of two plastid genes (*matK*, *rbcL*), two mitochondrial genes (*coxII*, *nad5*), six low-copy nuclear genes (*ADH*-like, *EF1A*, *H3*, *hsp90*, *RD19*-like, *SKP1*) and the 18S rDNA gene were constructed that incorporated the GS FLX Titanium forward primer A and a 10-bp multiplex identifier (MID) or reverse primer B (Table S4). The forward primer (Primer A-key) was: 5′-ccatctcatccctgcgtgtctccgactcag-MID-*template-specific-sequence*-3′; and the reverse primer (Primer B-key) was 5′-cctatcccctgtgtgccttggcagtctcag-*template-specific-sequence*-3′. MIDs 1 to 4 from the standard 454 set (Roche Technical Bulletin No. 013-2009) were selected, and each of the four species used was marked using a specific MID.

PCR amplification was conducted in a total volume of 50 µl, containing 1× High Fidelity PCR Buffer (Invitrogen), 5 mM of each deoxynucleoside triphosphate (Promega), 10 µM of each primer (Biolegio), 50 mM MgSO_4_, 1.0 U of Platinum®*Taq* High Fidelity, 20 µg BSA and 5 to 10 ng of total DNA using the following thermo profile: 94°C for 1 min (1 cycle); 94°C for 30 s, 55°C for 30 s and 68°C for 60 s (35 cycles); and then 68°C for 8 min (1 cycle).

Amplicons were purified using the Agencourt AMPure XP kit (Beckman Coultier) and quantified using the Quant-iT PicoGreen dsDNA Assay Kit (Invitrogen). Products from the same treatment (i.e. ‘fresh tissue’, ‘young herbarium’, or ‘old herbarium’) were mixed at equimolar concentrations to ca. 1*10^7^ molecules/µl. For each amplicon pool emPCR was performed following the emPCR Method Manual Lib-L for medium volume (Roche, 2009) and sequenced in a quarter of a PicoTiterPlate™ using a GS FLX Titanium Series. Pyrosequencing was carried out with primer A using the Genome Sequencer FLX (Roche Life Sciences Technology) at the Natural History Museum, University of Copenhagen, Denmark.

Amplicon sequence analysis was performed using the Galaxy platform [24], [25] and included the following: first, a plot reflecting the base quality distribution was constructed. For each treatment, raw 454 sequence data and quality scores were converted to FASTQ format [26]. Low quality 3-prime ends were trimmed using a fixed value of 5% of the sequence length. Clean reads were reconverted to FASTA format and filtered by MID identifiers, constructing one file for each species-treatment.

Subsequenctly, reads were mapped against a reference sequence under the Roche-454 98% identity criterion and only reporting matches above 90% of identity and covering at least 50% of the reference sequence. A BAM file was generated from the SAM output, to only report bases with at least 100× coverage. The filtered pile up was examined for regions with a drastic drop in coverage, such as at the end of a homopolymer or at the end of the sequence, and these regions were manually removed from the output.

In sequences of *EF1A*, *hsp90*, *H3*, and *SKP1* a minority of nucleotide positions were found to be polymorphic in fresh controls and were omitted from further analysis. We assumed these to result from either allelic variation or to be paralogous sequence variants (not shown).

Nucleotide substitutions were counted across reads and grouped into six substitution types that are effectively indistinguishable in PCR reactions [27]; (A→C/T→G), (A→G/T→C), (A→T/T→A), (C→A/G→T), (C→G/G→C) and (C→T/G→A). The numbers of observed substitutions among the herbarium data was scaled to match the total amount of nucleotides in data from fresh tissue. For example, the corrected young herbarium count of plastid data for substitution pair (A→G/T→C) was calculated as: (Observed young herbarium plastid A→G/T→C)*(Total nucleotides in data fresh tissue plastid DNA)/(Total nucleotides in data young herbarium plastid DNA) = (11634*10746437)/14193327 = 8808.65. Chi-square (χ^2^) tests of independence (at *p*≥0.05 level) were used to investigate whether the distributions of substitutions over the six substitution types were the same in sequence data obtained from fresh and herbarium DNA. The test was made two-sided because *a priori* the substitution direction from fresh DNA is not known. One-way analyses of variance (ANOVA) were performed to identify which of the six nucleotide substitution types occur at highest rates in each genomic compartment in herbarium DNA. For ANOVA, substitutions counts were expressed as numbers of substitutions per million total nucleotides observed (across reads).

## Supporting Information

Figure S1 DNA extraction gel images.
(DOCX)

Table S1 Plant specimens, specimen information and mean DNA yield.
(DOCX)

Table S2 PCR primers and corresponding amplification targets.
(DOCX)

Table S3 Real-time PCR primers and performance of standard curves.
(DOCX)

Table S4 Titanium fusion primers used for 454-sequencing.
(DOCX)

Table S5 Copy numbers of chloroplast, mitochondrial and nuclear gene amplicons in fresh and herbarium tissues.
(DOCX)

Table S6 Mean DNA yield and mean gene copy numbers for plastid, mitochondrial and nuclear DNA regions.
(DOCX)

Table S7 ANOVA on average number of miscoding lesions (per 10^6^ total nucleotides) for each substitution type across fresh and herbarium DNA. Separate analyses were performed for plastid, mitochondrial and nuclear DNA.
(DOCX)
