# Supplementary material for: DNA Damage in Plant Herbarium Tissue
Source: PLoS One. 2011 Dec 5;6(12):e28448. doi: 10.1371/journal.pone.0028448 (PMC3230621; doi:10.1371/journal.pone.0028448)
Supplement: Figure S1 — DNA extraction gel images. (DOCX) [file pone.0028448.s001.docx]

**Figure S1: DNA extraction gel images**

**
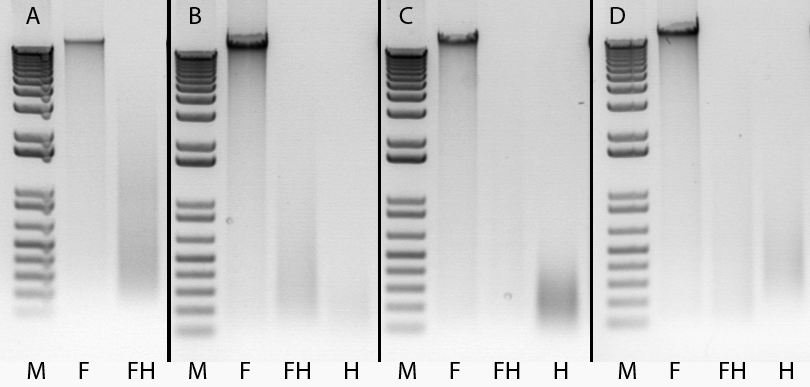
**

**Agarose gels containing DNA extracts from *Lonicera maackii* (A), *Ginkgo biloba* (B), *Laburnum anagyroides* (C) and *Liriodendron tulipifera* (D) of fresh tissue (F), young herbarium 8-7-2010 (FH) and old herbarium > 65 yrs. (H). M = 1 Kb Plus DNA Ladder (Invitrogen).**
