# Supplementary material for: DNA Damage in Plant Herbarium Tissue
Source: PLoS One. 2011 Dec 5;6(12):e28448. doi: 10.1371/journal.pone.0028448 (PMC3230621; doi:10.1371/journal.pone.0028448)
Supplement: Table S1 — Plant specimens, specimen information and mean DNA yield. (DOCX) [file pone.0028448.s002.docx]

**Table S1: Plant specimens, specimen information and mean DNA yield.**

| **Species, type of material** | **Herbarium collection number** | **Garden collection number/reference** | **Collection date** | **Remark** | **Mean DNA yield (ng DNA/mg DW)** |
| --- | --- | --- | --- | --- | --- |
| *Ginkgo biloba*, old herbarium | s.n. | Hortus Leiden | 1-08-1904 | Herbarium Lugd. Bat. No. 904320-3 | 33.91 |
| *Ginkgo biloba*, young herbarium | MS13 | Hortus Leiden | 8-7-2010 |  | 46.01 |
| *Ginkgo biloba*, fresh tissue |  | Hortus Leiden | 8-7-2010 |  | 394.83 |
| *Liriodendron tulipifera*, old herbarium | s.n. | Entrance | 28-06-1897 | Collected by J.W.C. Goedhart No. 908.126-236 | 60.70 |
| *Liriodendron tulipifera*, young herbarium | MS17 | Entrance | 8-7-2010 |  | 64.25 |
| *Liriodendron tulipifera*, fresh tissue |  | Entrance | 8-7-2010 |  | 152.43 |
| *Laburnum anagyroides*, old herbarium | s.n. | D15002 | 17-05-1946 | Collected by S.J. van Oostrom No. 94771-197 | 61.06 |
| *Laburnum anagyroides*, young herbarium | MS15 | D15002 | 8-7-2010 |  | 37.97 |
| *Laburnum anagyroides*, fresh tissue |  | D15002 | 8-7-2010 |  | 109.06 |
| *Lonicera maackii*, young herbarium | MS10 | 19.982 | 8-7-2010 |  | 103.40 |
| *Lonicera maackii*, fresh tissue |  | 19.982 | 8-7-2010 |  | 181.45 |
