# Supplementary material for: DNA Damage in Plant Herbarium Tissue
Source: PLoS One. 2011 Dec 5;6(12):e28448. doi: 10.1371/journal.pone.0028448 (PMC3230621; doi:10.1371/journal.pone.0028448)
Supplement: Table S2 — PCR primers and corresponding amplification targets. (DOCX) [file pone.0028448.s003.docx]

**Table S2: PCR primers and corresponding amplification targets**

| **Target species** | **Target region** | **Primer name** | **Primer sequence (5’---3’)** | **Reference homolog^A^** | **Reference gene (sub-)family^B^** |
| --- | --- | --- | --- | --- | --- |
| All four species | Chloroplast gene; Ribulose bisphosphate carboxylase, large subunit (*rbcL*) | rbcL_uniF | GACAACTGTKTGGACCGATG | X03853 | ORTHO001421 |
| All four species | Chloroplast gene; Ribulose bisphosphate carboxylase, large subunit (*rbcL*) | rbcL_uniR | TGCTTCGGCACAAAAYAAGA | X03853 | ORTHO001421 |
| All four species | Mitochondrial gene; NADH:ubiquinone oxidoreductase, chain 5 (*nad5*) | nad5_uniF | TTGGTGCTGYTGGRAAATCT | Y12731 | ORTHO012365 |
| All four species | Mitochondrial gene; NADH:ubiquinone oxidoreductase, chain 5 (*nad5*) | nad5_uniR | CATGAGCATCATRGCATAGG | Y12731 | ORTHO012365 |
| All four species | Nuclear gene; 18S ribosomal RNA (18S rRNA) | 18S_uniF | CTTCGGGAYCGGAGTAATGA | X02623 |  |
| All four species | Nuclear gene; 18S ribosomal RNA (18S rRNA) | 18S_uniR | TTAGCAGGCTGAGGTCTCGT | X02623 |  |
| *Ginkgo biloba* | Chloroplast gene; Maturase (*matK*) | matK_GikF | TTGATGCGATCGAAACATTC | AF279806 | ORTHO008814 |
| *Ginkgo biloba* | Chloroplast gene; Maturase (*matK*) | matK_GikR | GGAGTGAGCACCCTTCAGAG | AF279806 | ORTHO008814 |
| *Ginkgo biloba* | Mitochondrial gene; Cytochrome C oxidase subunit II (*coxII*) | COXII_GikF | GGTCCTCGTATCACGGATGT | AJ874265 | ORTHO007268 |
| *Ginkgo biloba* | Mitochondrial gene; Cytochrome C oxidase subunit II (*coxII*) | COXII_GikR | GAGGGATGTGTACCTCCAACA | AJ874265 | ORTHO007268 |
| *Ginkgo biloba* | Nuclear gene; Translation elongation factor EF1A (*EF1A*) | EF_GikF | GCGATCCACAAACCTTGATT | DR065003 | ORTHO000131 |
| *Ginkgo biloba* | Nuclear gene; Translation elongation factor EF1A (*EF1A*) | EF_GikR | GCCTATCCACCTTGGTCAAA | DR065003 | ORTHO000131 |
| *Ginkgo biloba* | Nuclear gene; Heat shock protein 90 (*hsp90*) | HSP90_GikF | AAGAGGGCACCTTTCGATCT | EX931765 | ORTHO000173 |
| *Ginkgo biloba* | Nuclear gene; Heat shock protein 90 (*hsp90*) | HSP90_GikR | AGAGTTCTCCACGGCCTTTT | EX931765 | ORTHO000173 |
| *Ginkgo biloba* | Nuclear gene; Peptidase C1A, papain (*RD19*-like) | RD19_GikF | TGATCCTTCAGACCCAAGAGC | EX935626 | ORTHO001008 |
| *Ginkgo biloba* | Nuclear gene; Peptidase C1A, papain (*RD19*-like) | RD19_GikR | TCAATGCTTAAGAAAGCTTAGCC | EX935626 | ORTHO001008 |
| *Ginkgo biloba* | Nuclear gene; S-phase kinase-associated protein (*SKP*1) | SKP1_GikF | TCCAGCTCCAACTCCAACTC | EX935761 | ORTHO001164 |
| *Ginkgo biloba* | Nuclear gene; S-phase kinase-associated protein (*SKP*1) | SKP1_GikR | CGCGTCATCAATTCATTTTG | EX935761 | ORTHO001164 |
| *Laburnum anagyroides* | Chloroplast gene; Maturase (*matK*) | matK_LabF | AGCGTTCTTTTTGAACGAATC | EU025898 | ORTHO008814 |
| *Laburnum anagyroides* | Chloroplast gene; Maturase (*matK*) | matK_labR | GCCCAAACCGGCTTACTAAT | EU025898 | ORTHO008814 |
| *Laburnum anagyroides* | Mitochondrial gene; Cytochrome C oxidase subunit II (*coxII*) | cox2_F | CGCTTTATGGCATTTCCAYT | AJ414385 | ORTHO007268 |
| *Laburnum anagyroides* | Mitochondrial gene; Cytochrome C oxidase subunit II (*coxII*) | cox2_R | CGACCAGGTACAGCATCACA | AJ414385 | ORTHO007268 |
| *Laburnum anagyroides* | Nuclear gene; Histone H3 (*H3*) | H3_LabF | TGGCTCGTACTAAGCAAACC | CA409775 | ORTHO000105 |
| *Laburnum anagyroides* | Nuclear gene; Histone H3 (*H3*) | H3_LabR | CGAATACGCCTAGCGAGTTG | CA409775 | ORTHO000105 |
| *Laburnum anagyroides* | Nuclear gene; Heat shock protein 90 (*hsp90*) | HSP90_Labf | GCTTTACGAGGCTTTCTCCA | GW583316 | ORTHO000173 |
| *Laburnum anagyroides* | Nuclear gene; Heat shock protein 90 (*hsp90*) | HSP90_LabR | GTACCCAGCCATGCTGCTAT | GW583316 | ORTHO000173 |
| *Laburnum anagyroides* | Nuclear gene; S-phase kinase-associated protein (*SKP*1) | SKP1_LabF | CAACACCACCAAAGAAGATCA | FG093018 | ORTHO001164 |
| *Laburnum anagyroides* | Nuclear gene; S-phase kinase-associated protein (*SKP*1) | SKP1_LabR | CCACTGGTTTTCCCTACGAA | FG093018 | ORTHO001164 |
| *Liriodendron tulipifera* | Chloroplast gene; Maturase (*matK*) | matK_LirF | GGGCGCAACAAGAGTTTTTA | AF123480 | ORTHO008814 |
| *Liriodendron tulipifera* | Chloroplast gene; Maturase (*matK*) | matK_LirR | TCGCTCAAGAAAAGTTCCAGA | AF123480 | ORTHO008814 |
| *Liriodendron tulipifera* | Mitochondrial gene; Cytochrome C oxidase subunit II (*coxII*) | COX2_LirF | GATCTCAAGACGCAGCAACA | AY832090 | ORTHO007268 |
| *Liriodendron tulipifera* | Mitochondrial gene; Cytochrome C oxidase subunit II (*coxII*) | COX2_LirR | TCCTATGCTGGGAGCATTTC | AY832090 | ORTHO007268 |
| *Liriodendron tulipifera* | Nuclear gene; Alcohol dehydrogenase (*ADH*) | ADH_LirF | AGGGTGTGACGGATCTCGAACC | CK743816 | ORTHO000373 |
| *Liriodendron tulipifera* | Nuclear gene; Alcohol dehydrogenase (*ADH*) | ADH_LirR | AAGCCCCGGAGATCCTTG | CK743816 | ORTHO000373 |
| *Liriodendron tulipifera* | Nuclear gene; Translation elongation factor EF1A (*EF1A*) | EF_LirF | TATTTCAGGATTTGAGGGTGACAAC | FD500602 | ORTHO000131 |
| *Liriodendron tulipifera* | Nuclear gene; Translation elongation factor EF1A (*EF1A*) | EF_LirR | GCACTGGAGCATACCCATTT | FD500602 | ORTHO000131 |
| *Liriodendron tulipifera* | Nuclear gene; Heat shock protein 90 (*hsp90*) | HSP90_LirF | CTGGATGAGGAAGCCAGAAG | FD491900 | ORTHO000173 |
| *Liriodendron tulipifera* | Nuclear gene; Heat shock protein 90 (*hsp90*) | HSP90_LirR | CCGCTCTTGGTGGAGTGA | FD491900 | ORTHO000173 |
| *Liriodendron tulipifera* | Nuclear gene; Peptidase C1A, papain (*RD19*-like) | RD19_LirF | TGCGATACATCTGACCCAAG | FD498985 | ORTHO001008 |
| *Liriodendron tulipifera* | Nuclear gene; Peptidase C1A, papain (*RD19*-like) | RD19_LirR | GTGACCCCTGCAAATCTTGT | FD498985 | ORTHO001008 |
| *Lonicera maackii* | Mitochondrial gene; Cytochrome C oxidase subunit II (*coxII*) | cox2_F | CGCTTTATGGCATTTCCAYT | AJ414385 | ORTHO007268 |
| *Lonicera maackii* | Mitochondrial gene; Cytochrome C oxidase subunit II (*coxII*) | cox2_R | CGACCAGGTACAGCATCACA | AJ414385 | ORTHO007268 |
| *Lonicera maackii* | Nuclear gene; Translation elongation factor EF1A (*EF1A*) | Ef1_OrF | CCCAATCTCTGGATTTGAGG | EY827912 | ORTHO000131 |
| *Lonicera maackii* | Nuclear gene; Translation elongation factor EF1A (*EF1A*) | Ef1_orR | CGGGATCATCCTTGGAGTTA | EY827912 | ORTHO000131 |
| *Lonicera maackii* | Nuclear gene; Heat shock protein 90 (*hsp90*) | HSP90_OrF | GTCATCCGCAAGAACTTGGT | EY767013 | ORTHO000173 |
| *Lonicera maackii* | Nuclear gene; Heat shock protein 90 (*hsp90*) | HSP90_OrR | AACACGGTCAGACACCACAA | EY767013 | ORTHO000173 |
| *Lonicera maackii* | Nuclear gene; S-phase kinase-associated protein (*SKP*1) | SKP1_OrF | GACGGTGAGRCGTTCGAGGT | FJ851401 | ORTHO001164 |
| *Lonicera maackii* | Nuclear gene; S-phase kinase-associated protein (*SKP*1) | SKP1_OrR | GAAGTCATTCTTGATGTTGAARGT | FJ851401 | ORTHO001164 |

A: Reference sequence used for PCR primer construction.

B: Gene families as delineated by PLAZA 2.0 (Proost et al. 2009).
