# Supplementary material for: DNA Damage in Plant Herbarium Tissue
Source: PLoS One. 2011 Dec 5;6(12):e28448. doi: 10.1371/journal.pone.0028448 (PMC3230621; doi:10.1371/journal.pone.0028448)
Supplement: Table S3 — Real-time PCR primers and performance of standard curves. (DOCX) [file pone.0028448.s004.docx]

**Table S3: Real-time PCR primers and performance of standard curves.**

| **Target species** | **Target region^A^** | **Primer name** | **Primer sequence (5’---3’)** | **Standard curve^B^**  **(R^2^ value)** | **Amplification efficiency^c^** |
| --- | --- | --- | --- | --- | --- |
| Exogenous control | *Wg* | wg_Qf | CCGGGTGTCAAAGACTTAGTG | y = -3,1195x + 35,418 (0.99) | 2.09 |
| Exogenous control | *Wg* | wg_Qr | GTCGACTCCGATGCTCGTAT |  |  |
| *G. biloba* | *coxII* | 2Gik_Qcox2_F | GAGGGTGATGGAAGTGCAAC | y = -3,2508x + 36,154 (0.99) | 2.03 |
| *G. biloba* | *coxII* | 2Gik_Qcox2_R | TACGGTCCCTCGAGTCTCTC |  |  |
| *G. biloba* | *EF1A* | 3GIK_QEF_F | CACCATGAAGCACTCCAAGA | y = -3,4097x + 36,357 (0.99) | 1.96 |
| *G. biloba* | *EF1A* | 3GIK_QEF_R | GTGAAGTTTGCAGCCTCCTT |  |  |
| *G. biloba* | *hsp90* | 3Gik_QHSP90_F | AAGAGGGCACCTTTCGATCT | y = -3,352x + 35,596 (0.99) | 1.99 |
| *G. biloba* | *hsp90* | 3Gik_QHSP90_R | TTCGGGAATCAACTCCTCAC |  |  |
| *G. biloba* | *matK* | Gik_QmatK_F | CGCTCCGGAAGGAAATAC | y = -3,4370x + 36,355 (0.99) | 1.95 |
| *G. biloba* | *matK* | Gik_QmatK_R | GTTTCCGAAGGGGAACTAGG |  |  |
| *G. biloba* | *nad5* | Gik_Qnad5_F | CTTCTGCAGGAGCTATGACG | y = -3,3779x + 37,686 (0.99) | 1.98 |
| *G. biloba* | *nad5* | Gik_Qnad5_R | CGCGAGCAGGGATCATATAG |  |  |
| *G. biloba* | *rbcL* | Gik_QrbcL_F | ACGAGCTCTACGTCTGGAAG | y = -3,193x + 34,936 (0.99) | 2.06 |
| *G. biloba* | *rbcL* | Gik_QrbcL_R | GGGGACGGCCATATTTATTC |  |  |
| *L. anagyroides* | *coxII* | Lab_Qcox2_F | CAGTTCCGATGAACAGTCACTC | y = -3,5015x + 37,233 (0.99) | 1.93 |
| *L. anagyroides* | *coxII* | Lab_Qcox2_R | TTTGGCTGGTACAACCACTC |  |  |
| *L. anagyroides* | *hsp90* | 2Lab_QHSP90_F | CTCGGATGAAGGAAGGTCAG | y = -3,5665x + 37,768 (0.99) | 1.91 |
| *L. anagyroides* | *hsp90* | 2Lab_QHSP90_R | CAATGGCGTCAACCATGTAG |  |  |
| *L. anagyroides* | *matK* | Lab_QmatK_F | ACCAATTCTCCGAGCGTTCA | y = -3,2413x + 36,621 (0.99) | 2.03 |
| *L. anagyroides* | *matK* | Lab_QmatK_R | TCCAGCATTTGACTCCGTACC |  |  |
| *L. anagyroides* | *nad5* | 2Lab_Qnad5_F | AGGGTCCCACTCCAGTATCC | y = -3,4378x + 38,061 (0.99) | 1.95 |
| *L. anagyroides* | *nad5* | 2Lab_Qnad5_R | CAAAGCCGTAGGTGGGTATTC |  |  |
| *L. anagyroides* | *rbcL* | Lab_QrbcL_F | AGTATGGCCGTCCCCTATTG | y = -3,4002x + 37,197 (0.99) | 1.97 |
| *L anagyroides* | *rbcL* | Lab_QrbcL_R | AGTCCACCGCGTAGACATTC |  |  |
| *L. anagyroides* | *SKP1* | 2Lab_QSKP1_F | TCCCTCTTCCTAACGTCACC | y = -3,4773x + 37,152 (0.99) | 1.94 |
| *L. anagyroides* | *SKP1* | 2Lab_QSKP1_R | CGTCCCAAGCCTTTAGATCC |  |  |
| *L. tulipifera* | *ADH* | 3Lir_QADH_F | ATGGACAGTCGAGGTTCTCC | y = -3,5797x + 37,167 (0.99) | 1.90 |
| *L. tulipifera* | *ADH* | 3Lir_QADH_R | AGGGTTGATCTTGGCAACAC |  |  |
| *L. tulipifera* | *coxII* | 2Lir_Qcox2_F | TCATCCCGATGTTCATTGC | y = -3,2639x + 35,792 (0.99) | 2.02 |
| *L. tulipifera* | *coxII* | 2Lir_Qcox2_R | CGCACTCCGATACCATTGAT |  |  |
| *L. tulipifera* | *EF1A* | 3Lir_QEF_F | TGGTCGTGTTGAGACTGGAG | y = -3,5527x + 36,962 (0.99) | 1.91 |
| *L. tulipifera* | *EF1A* | 3Lir_QEF_R | GCCAACATTGTCACCTGGTA |  |  |
| *L. tulipifera* | *hsp90* | 2Lir_Qhsp90_F | ATGATTGGGAGGAGCATCTG | y = -3,2276x + 35,801 (0.98) | 2.04 |
| *L. tulipifera* | *hsp90* | 2Lir_Qhsp90_R | GCTTGATGTTGTTGGGCTTC |  |  |
| *L. tulipifera* | *matK* | 2Lir_QmatK_F | ACGATCAACATCCTCTGGAAC | y = -3,3589x + 36,855 (0.99) | 1.98 |
| *L. tulipifera* | *matK* | 2Lir_QmatK_R | GCATGAAAGGGTCCTTGAAC |  |  |
| *L. tulipifera* | *nad5* | Lir_Qnad5_F | ATACTCGGTCACCCGATGC | y = -3,3159x + 36,645 (0.92) | 2.00 |
| *L. tulipifera* | *nad5* | Lir_Qnad5_R | AGGGGAGCACCTTGCTATC |  |  |
| *L. tulipifera* | *rbcL* | Lir_QrbcL_F | ACGAGCTCTACGTCTGGAAG | y = -3,3095x + 37,185 (0.99) | 2.01 |
| *L. tulipifera* | *rbcL* | Lir_QrbcL_R | GGGGACGACCATACTTGTTC |  |  |
| *L. maackii* | *coxII* | 2or_Qcox2_F | CGATTCCAGAAGATGATCCAG | y = -3,3316x + 36,627 (0.99) | 2.00 |
| *L. maackii* | *coxII* | 2or_Qcox2_R | TGAGGAAGGTACAGCCCAAC |  |  |
| *L. maackii* | *hsp90* | 2Or_QHSP90_F | GCAAGAAGGCAGTGGAGAAC | y = -3,2148x + 35,798 (0.99) | 2.05 |
| *L. maackii* | *hsp90* | 2Or_QHSP90_r | TGCCCTCAAACTCCTTCAAC |  |  |
| *L. maackii* | *nad5* | 2or_Qnad5_f | AGGGTCCCACTCCAGTATCC | y = -3,2323x + 36,138 (0.99) | 2.04 |
| *L. maackii* | *nad5* | 2or_Qnad5_R | CAAAGCCGTAGGTGGGTATTC |  |  |
| *L. maackii* | *rbcL* | 2or_QrbcL_F | CCGTTGCTGGAGAAGAAACT | y = -3,3989x + 36,543 (0.99) | 1.97 |
| *L. maackii* | *rbcL* | 2or_QrbcL_R | CAGGGCTTTGAACCCAAAT |  |  |
| *L. maackii* | *SKP1* | 2or_QSKP1_F | ACTGCAAGAAGCACGTGGA | y = -3,2918x + 36,696 (0.99) | 2.01 |
| *L. maackii* | *SKP1* | 2or_QSKP1_R | GATCGAACAAAGTCCCCTGA |  |  |

A: Target region abbreviations refer to gene descriptions in Table S2.

B: Linear trend lines represent the least square fit through triplicate measurements.

C: Amplification efficiency = 10^(-1/slope)^
