# Supplementary material for: DNA Damage in Plant Herbarium Tissue
Source: PLoS One. 2011 Dec 5;6(12):e28448. doi: 10.1371/journal.pone.0028448 (PMC3230621; doi:10.1371/journal.pone.0028448)
Supplement: Table S4 — Titanium fusion primers used for 454-sequencing. (DOCX) [file pone.0028448.s005.docx]

**Table S4: Titanium fusion primers used for 454-sequencing.**

| **Target species** | **Target region^A^** | **Primer name** | **Primer sequence (5’---3’)** | **Product length (bp)** |
| --- | --- | --- | --- | --- |
| *G. biloba* | *coxII* | 454COXII_GikF | ccatctcatccctgcgtgtctccgactcagACGCTCGACA*AAAGCTATTGGACATCAACGGTA* | 362 bp |
| *G. biloba* | *coxII* | 454COXII_GikR | cctatcccctgtgtgccttggcagtctcag*GAGGGATGTGTACCTCCAACA* |  |
| *G. biloba* | *EF1A* | 454EF_GikF | ccatctcatccctgcgtgtctccgactcagACGCTCGACA*GCGATCCACAAACCTTGATT* | 366 bp |
| *G. biloba* | *EF1A* | 454EF_GikR | cctatcccctgtgtgccttggcagtctcag*TCTTGACATTGAAGCCCACA* |  |
| *G. biloba* | *hsp90* | 454HSP90_Gikf | ccatctcatccctgcgtgtctccgactcagACGCTCGACA*CGTGAAATGCTCCAACAGAA* | 388 bp |
| *G. biloba* | *hsp90* | 454HSP90_GikR | cctatcccctgtgtgccttggcagtctcag*AGAGTTCTCCACGGCCTTTT* |  |
| *G. biloba* | *matK* | 454matK_GikF | ccatctcatccctgcgtgtctccgactcagACGCTCGACA*TTGATGCGATCGAAACATTC* | 375 bp |
| *G. biloba* | *matK* | 454matK_GikR3 | cctatcccctgtgtgccttggcagtctcag*TTCCACAGAAACAGAAAGAACT* |  |
| *G. biloba* | *nad5* | 454nad5_GikF | ccatctcatccctgcgtgtctccgactcagACGCTCGACA*CCACGGCTTCGATTGTTATT* | 384 bp |
| *G. biloba* | *nad5* | 454nad5_uniR | cctatcccctgtgtgccttggcagtctcag*CATGAGCATCATRGCATAGG* |  |
| *G. biloba* | *rbcL* | 454rbcL_GikF | ccatctcatccctgcgtgtctccgactcagACGCTCGACA*GACAACTGTKTGGACCGATG* | 378 bp |
| *G. biloba* | *rbcL* | 454rbcL_GikR | cctatcccctgtgtgccttggcagtctcag*GGGGACGGCCATATTTATTC* |  |
| *G. biloba* | *RD19*-like | 454_RD19_GikF | ccatctcatccctgcgtgtctccgactcagACGCTCGACA*GCCTCTATCGGGTAAGTGAA* | 270 bp |
| *G. biloba* | *RD19*-like | 454_RD19_GikR | cctatcccctgtgtgccttggcagtctcag*CAGTCCTACCTATTTTGCACCCTTA* |  |
| *G. biloba* | *SKP1* | 454SKP1_GikF | ccatctcatccctgcgtgtctccgactcagACGCTCGACA*GAAGGCTAGTTGTTTCTAGGTTTCT* | 379 bp |
| *G. biloba* | *SKP1* | 454SKP1_GikR | cctatcccctgtgtgccttggcagtctcag*CCGGAGTCTTCCCCTTTATC* |  |
| *G. biloba* | 18S rRNA | 454_18S_GikF | ccatctcatccctgcgtgtctccgactcagACGCTCGACA*CATAAACGATGCCGACYAG* | 394 bp |
| *G. biloba* | 18S rRNA | 454_18S_uniR | cctatcccctgtgtgccttggcagtctcag*TTAGCAGGCTGAGGTCTCGT* |  |
| *L. anagyroides* | *coxII* | 454cox2_LABF | ccatctcatccctgcgtgtctccgactcagACGAGTGCGT*CGCTTTATGGCATTTCCAYT* | 505 bp |
| *L. anagyroides* | *coxII* | 454cox2_LABR | cctatcccctgtgtgccttggcagtctcag*CGACCAGGTACAGCATCACA* |  |
| *L. anagyroides* | *H3* | 454H3_LabF | ccatctcatccctgcgtgtctccgactcagACGAGTGCGT*TGTTGCAAGCCTGCTACTGA* | 477 bp |
| *L. anagyroides* | *H3* | 454H3_LabR | cctatcccctgtgtgccttggcagtctcag*GCAACAGTTCCAGGACGGTA* |  |
| *L. anagyroides* | *hsp90* | 454HSP90_labF2 | ccatctcatccctgcgtgtctccgactcagACGAGTGCGT*CGGATGAAGGAAGGTCAGA* | 505 bp |
| *L. anagyroides* | *hsp90* | 454HSP90_LABR | cctatcccctgtgtgccttggcagtctcag*GTACCCAGCCATGCTGCTAT* |  |
| *L. anagyroides* | *matK* | 454matK_LABF | ccatctcatccctgcgtgtctccgactcagACGAGTGCGT*AGCGTTCTTTTTGAACGAATC* | 537 bp |
| *L. anagyroides* | *matK* | 454matK_LABR | cctatcccctgtgtgccttggcagtctcag*GCCCAAACCGGCTTACTAAT* |  |
| *L. anagyroides* | *nad5* | 454nad5_LABF | ccatctcatccctgcgtgtctccgactcagACGAGTGCGT*TTGGTGCTGYTGGRAAATCT* | 543 bp |
| *L. anagyroides* | *nad5* | 454nad5_uniR | cctatcccctgtgtgccttggcagtctcag*CATGAGCATCATRGCATAGG* |  |
| *L. anagyroides* | *rbcL* | 454rbcL_LABF | ccatctcatccctgcgtgtctccgactcagACGAGTGCGT*GACAACTGTKTGGACCGATG* | 545 bp |
| *L. anagyroides* | *rbcL* | 454rbcL_uniR | cctatcccctgtgtgccttggcagtctcag*TGCTTCGGCACAAAAYAAGA* |  |
| *L. anagyroides* | *SKP1* | 454SKP1_LabF | ccatctcatccctgcgtgtctccgactcagACGAGTGCGT*GACGCTGATTTCGTCAAGGT* | 508 bp |
| *L. anagyroides* | *SKP1* | 454SKP1_LabR | cctatcccctgtgtgccttggcagtctcag*CACAGCACCAGTCAGTGTCA* |  |
| *L. anagyroides* | 18S rRNA | 454_18S_LABF | ccatctcatccctgcgtgtctccgactcagACGAGTGCGT*CTTCGGGAYCGGAGTAATGA* | 574bp |
| *L. anagyroides* | 18S rRNA | 454_18S_uniR | cctatcccctgtgtgccttggcagtctcag*TTAGCAGGCTGAGGTCTCGT* |  |
| *L. tulipifera* | *ADH* | 454_ADH_LirF | ccatctcatccctgcgtgtctccgactcagAGACGCACTC*GGGAAGCCCATCTACCATTT* | 464 bp |
| *L. tulipifera* | *ADH* | 454_ADH_LirR | cctatcccctgtgtgccttggcagtctcag*ATTCCCTCGGCAAAGAAGAT* |  |
| *L. tulipifera* | *coxII* | 454COX2_LirF | ccatctcatccctgcgtgtctccgactcagAGACGCACTC*GATCTCAAGACGCAGCAACA* | 562 bp |
| *L. tulipifera* | *coxII* | 454COX2_LirR | cctatcccctgtgtgccttggcagtctcag*TCCTATGCTGGGAGCATTTC* |  |
| *L. tulipifera* | *EF1A* | 454EF_LirF | ccatctcatccctgcgtgtctccgactcagAGACGCACTC*TATTTCAGGATTTGAGGGTGACAAC* | 540 bp |
| *L. tulipifera* | *EF1A* | 454EF_LirR | cctatcccctgtgtgccttggcagtctcag*GCACTGGAGCATACCCATTT* |  |
| *L. tulipifera* | *hsp90* | 454HSP90_LirF | ccatctcatccctgcgtgtctccgactcagAGACGCACTC*CTGGATGAGGAAGCCAGAAG* | 595 bp |
| *L. tulipifera* | *hsp90* | 454HSP90_LirR | cctatcccctgtgtgccttggcagtctcag*CCGCTCTTGGTGGAGTGA* |  |
| *L. tulipifera* | *matK* | 454matK_LirF | ccatctcatccctgcgtgtctccgactcagAGACGCACTC*GGGCGCAACAAGAGTTTTTA* | 550 bp |
| *L. tulipifera* | *matK* | 454matK_LirR | cctatcccctgtgtgccttggcagtctcag*TCGCTCAAGAAAAGTTCCAGA* |  |
| *L. tulipifera* | *nad5* | 454nad5_LirF | ccatctcatccctgcgtgtctccgactcagAGACGCACTC*TTGGTGCTGYTGGRAAATCT* | 543 bp |
| *L. tulipifera* | *nad5* | 454nad5_uniR | cctatcccctgtgtgccttggcagtctcag*CATGAGCATCATRGCATAGG* |  |
| *L. tulipifera* | *rbcL* | 454rbcL_LirF | ccatctcatccctgcgtgtctccgactcagAGACGCACTC*GACAACTGTKTGGACCGATG* | 545 bp |
| *L. tulipifera* | *rbcL* | 454rbcL_uniR | cctatcccctgtgtgccttggcagtctcag*TGCTTCGGCACAAAAYAAGA* |  |
| *L. tulipifera* | *RD19*-like | 454RD19_LirF | ccatctcatccctgcgtgtctccgactcagAGACGCACTC*CCCTCTTGCAGGTAAGCA* | 464 bp |
| *L. tulipifera* | *RD19*-like | 454RD19_LirR | cctatcccctgtgtgccttggcagtctcag*CACAGCCCTCCCTCAATAC* |  |
| *L. tulipifera* | 18S rRNA | 454_18S_LirF | ccatctcatccctgcgtgtctccgactcagAGACGCACTC*CTTCGGGAYCGGAGTAATGA* | 574 bp |
| *L. tulipifera* | 18S rRNA | 454_18S_uniR | cctatcccctgtgtgccttggcagtctcag*TTAGCAGGCTGAGGTCTCGT* |  |
| *L. maackii* | *coxII* | 454cox2_OrF | ccatctcatccctgcgtgtctccgactcagAGCACTGTAG*CAATGGACGAGGTAGTAGTAGATCC* | 365 bp |
| *L. maackii* | *coxII* | 454cox2_OrR | cctatcccctgtgtgccttggcagtctcag*CGACCAGGTACAGCATCACA* |  |
| *L. maackii* | *EF1A* | 454Ef1_OrF | ccatctcatccctgcgtgtctccgactcagAGCACTGTAG*CCCAATCTCTGGATTTGAGG* | 275 bp |
| *L. maackii* | *EF1A* | 454Ef1_orR | cctatcccctgtgtgccttggcagtctcag*GGTACCGGTTTCAACACGTC* |  |
| *L. maackii* | *hsp90* | 454HSP90_OrF | ccatctcatccctgcgtgtctccgactcagAGCACTGTAG*GTCATCCGCAAGAACTTGGT* | 281 bp |
| *L. maackii* | *hsp90* | 454HSP90_OrR2 | cctatcccctgtgtgccttggcagtctcag*CATAATCCTTCAGGCTGGTCA* |  |
| *L. maackii* | *nad5* | 454nad5_OrF | ccatctcatccctgcgtgtctccgactcagAGCACTGTAG*TTCAACTTGCAGTCAATTAGGC* | 284 bp |
| *L. maackii* | *nad5* | 454nad5_uniR | cctatcccctgtgtgccttggcagtctcag*CATGAGCATCATRGCATAGG* |  |
| *L. maackii* | *rbcL* | 454rbcL_OrF | ccatctcatccctgcgtgtctccgactcagAGCACTGTAG*GACAACTGTKTGGACCGATG* | 375 bp |
| *L. maackii* | *rbcL* | 454rbcL_OrR | cctatcccctgtgtgccttggcagtctcag*GGCGACCGTACTTGTTCAAT* |  |
| *L. maackii* | *SKP1* | 454SKP1_OrF | ccatctcatccctgcgtgtctccgactcagAGCACTGTAG*TACTGCAAGAAGCACGTGGA* | 380 bp |
| *L. maackii* | *SKP1* | 454SKP1_OrR | cctatcccctgtgtgccttggcagtctcag*TCACAAATAACAACAAAAGTTCCT* |  |
| *L. maackii* | 18S rRNA | 454_18S_OrF | ccatctcatccctgcgtgtctccgactcagAGCACTGTAG*AACTTAAAGGAATTGACGGAAGG* | 284 bp |
| *L. maackii* | 18S rRNA | 454_18S_uniR | cctatcccctgtgtgccttggcagtctcag*TTAGCAGGCTGAGGTCTCGT* |  |

A: Target region abbreviations refer to gene descriptions in Table S2.
