# Supplementary material for: DNA Damage in Plant Herbarium Tissue
Source: PLoS One. 2011 Dec 5;6(12):e28448. doi: 10.1371/journal.pone.0028448 (PMC3230621; doi:10.1371/journal.pone.0028448)
Supplement: Table S5 — Copy numbers of chloroplast, mitochondrial and nuclear gene amplicons in fresh and herbarium tissues. (DOCX) [file pone.0028448.s006.docx]

**Table S5: Copy numbers of chloroplast, mitochondrial and nuclear gene amplicons in fresh and herbarium tissues.**

| **Species, type of material** | **Target gene** | **Copy number /**  **ng total DNA** |
| --- | --- | --- |
| *G. biloba*, old herbarium | *rbcL* | 1869.48 |
| *G. biloba*, old herbarium | *matK* | 4331.55 |
| *G. biloba*, old herbarium | *coxII* | 546.78 |
| *G. biloba*, old herbarium | *nad5* | 600.75 |
| *G. biloba*, old herbarium | *EF1A* | 10.42 |
| *G. biloba*, old herbarium | *hsp90* | 10.66 |
| *G. biloba*, young herbarium (8-7-2010) | *rbcL* | 18708.01 |
| *G. biloba*, young herbarium (8-7-2010) | *matK* | 23328.19 |
| *G. biloba*, young herbarium (8-7-2010) | *coxII* | 4315.28 |
| *G. biloba*, young herbarium (8-7-2010) | *nad5* | 3582.06 |
| *G. biloba*, young herbarium (8-7-2010) | *EF1A* | 69.81 |
| *G. biloba*, young herbarium (8-7-2010) | *hsp90* | 53.96 |
| *G. biloba*, fresh tissue (8-7-2010) | *rbcL* | 149671.40 |
| *G. biloba*, fresh tissue (8-7-2010) | *matK* | 128558.43 |
| *G. biloba*, fresh tissue (8-7-2010) | *coxII* | 11115.22 |
| *G. biloba*, fresh tissue (8-7-2010) | *nad5* | 19159.63 |
| *G. biloba*, fresh tissue (8-7-2010) | *EF1A* | 995.85 |
| *G. biloba*, fresh tissue (8-7-2010) | *hsp90* | 657.60 |
| *L. anagyroides*, old herbarium | *rbcL* | 36570.49 |
| *L. anagyroides*, old herbarium | *matK* | 79844.09 |
| *L. anagyroides*, old herbarium | *coxII* | 4343.80 |
| *L. anagyroides*, old herbarium | *nad5* | 4269.68 |
| *L. anagyroides*, old herbarium | *SKP1* | 116.01 |
| *L. anagyroides*, old herbarium | *hsp90* | 61.03 |
| *L. anagyroides*, young herbarium (8-7-2010) | *rbcL* | 55101.29 |
| *L. anagyroides*, young herbarium (8-7-2010) | *matK* | 94534.47 |
| *L. anagyroides*, young herbarium (8-7-2010) | *coxII* | 6320.27 |
| *L. anagyroides*, young herbarium (8-7-2010) | *nad5* | 6266.08 |
| *L. anagyroides*, young herbarium (8-7-2010) | *SKP1* | 440.79 |
| *L. anagyroides*, young herbarium (8-7-2010) | *hsp90* | 326.81 |
| *L. anagyroides*, fresh tissue (8-7-2010) | *rbcL* | 372285.62 |
| *L. anagyroides*, fresh tissue (8-7-2010) | *matK* | 557852.30 |
| *L. anagyroides*, fresh tissue (8-7-2010) | *coxII* | 42892.58 |
| *L. anagyroides*, fresh tissue (8-7-2010) | *nad5* | 35263.57 |
| *L. anagyroides*, fresh tissue (8-7-2010) | *SKP1* | 1897.73 |
| *L. anagyroides*, fresh tissue (8-7-2010) | *hsp90* | 630.84 |
| *L. tulipifera*, old herbarium | *rbcL* | 53681.10 |
| *L. tulipifera*, old herbarium | *matK* | 39233.87 |
| *L. tulipifera*, old herbarium | *coxII* | 1101.51 |
| *L. tulipifera*, old herbarium | *nad5* | 1320.59 |
| *L. tulipifera*, old herbarium | *ADH* | 407.67 |
| *L. tulipifera*, old herbarium | *EF1A* | 21.34 |
| *L. tulipifera*, old herbarium | *hsp90* | 24.71 |
| *L. tulipifera*, young herbarium (8-7-2010) | *rbcL* | 88632.51 |
| *L. tulipifera*, young herbarium (8-7-2010) | *matK* | 57568.39 |
| *L. tulipifera*, young herbarium (8-7-2010) | *coxII* | 2221.42 |
| *L. tulipifera*, young herbarium (8-7-2010) | *nad5* | 3946.09 |
| *L. tulipifera*, young herbarium (8-7-2010) | *ADH* | 466.31 |
| *L. tulipifera*, young herbarium (8-7-2010) | *EF1A* | 51.91 |
| *L. tulipifera*, young herbarium (8-7-2010) | *hsp90* | 148.84 |
| *L. tulipifera*, fresh tissue (8-7-2010) | *rbcL* | 1095497.61 |
| *L. tulipifera*, fresh tissue (8-7-2010) | *matK* | 695469.75 |
| *L. tulipifera*, fresh tissue (8-7-2010) | *coxII* | 25815.51 |
| *L. tulipifera*, fresh tissue (8-7-2010) | *nad5* | 30098.75 |
| *L. tulipifera*, fresh tissue (8-7-2010) | *ADH* | 2954.92 |
| *L. tulipifera*, fresh tissue (8-7-2010) | *EF1A* | 542.15 |
| *L. tulipifera*, fresh tissue (8-7-2010) | *hsp90* | 1662.38 |
| *L. maackii,* young herbarium (8-7-2010) | *rbcL* | 27836.98 |
| *L. maackii,* young herbarium (8-7-2010) | *coxII* | 4020.89 |
| *L. maackii,* young herbarium (8-7-2010) | *nad5* | 4558.67 |
| *L. maackii,* young herbarium (8-7-2010) | *hsp90* | 434.43 |
| *L. maackii,* young herbarium (8-7-2010) | *SKP1* | 785.75 |
| *L. maackii,* fresh tissue (8-7-2010) | *rbcL* | 475749.48 |
| *L. maackii,* fresh tissue (8-7-2010) | *coxII* | 75394.25 |
| *L. maackii,* fresh tissue (8-7-2010) | *nad5* | 48209.09 |
| *L. maackii,* fresh tissue (8-7-2010) | *hsp90* | 2521.15 |
| *L. maackii,* fresh tissue (8-7-2010) | *SKP1* | 4628.39 |
