# Supplementary material for: DNA Damage in Plant Herbarium Tissue
Source: PLoS One. 2011 Dec 5;6(12):e28448. doi: 10.1371/journal.pone.0028448 (PMC3230621; doi:10.1371/journal.pone.0028448)
Supplement: Table S6 — Mean DNA yield and mean gene copy numbers for plastid, mitochondrial and nuclear DNA regions. (DOCX) [file pone.0028448.s007.docx]

**Table S6: Mean DNA yield and mean gene copy numbers for plastid, mitochondrial and nuclear DNA regions.**

| **Sample type** | **Mean DNA yield ± SE (ng/mg DW tissue)^1^** | **Mean gene copy numbers / ng total DNA ± SE** | | |
| --- | --- | --- | --- | --- |
|  |  | **Plastid** | **Mitochondrial** | **Nuclear** |
| Fresh tissue (8-7-2010) | 278.0 ± 25.6 (A)^2^ | 4.96E+05 ± 1.27E+05 | 3.60E+04 ± 7.06E+03 | 1.83E+03 ± 4.53E+02 |
| Young herbarium (8-7-2010) | 62.9 ± 8.2 (B) | 5.22E+04 ± 1.17E+04 | 4.40E+03 ± 4.81E+02 | 3.09E+02 ± 8.33E+01 |
| Old herbarium (>65 yrs.) | 51.9 ± 4.8 (B) | 3.59E+04 ± 1.21E+04 | 2.03E+03 ± 7.30E+02 | 9.31E+01 ± 5.43E+01 |

1: Mean values were statistically different (F(2,47) = 42.875, P < 0.001).

2: Values statistically different in Games-Howell post-hoc test are indicated in brackets by different letters (A or B).
