# Supplementary material for: DNA Damage in Plant Herbarium Tissue
Source: PLoS One. 2011 Dec 5;6(12):e28448. doi: 10.1371/journal.pone.0028448 (PMC3230621; doi:10.1371/journal.pone.0028448)
Supplement: Table S7 — ANOVA on average number of miscoding lesions (per 106 total nucleotides) for each substitution type across fresh and herbarium DNA. Separate analyses were performed for plastid, mitochondrial and nuclear DNA. (DOCX) [file pone.0028448.s008.docx]

**Table S7: ANOVA on average number of miscoding lesions (per 10^6^ total nucleotides) for each substitution type across fresh and herbarium DNA. Separate analyses were performed for plastid, mitochondrial and nuclear DNA.**

| **DNA type** | **Substitution type** | **Fresh tissue** | **Young herbarium** | **Old herbarium** | **ANOVA (*P*-value)** |
| --- | --- | --- | --- | --- | --- |
| **Plastid** | **(A→G/T→C)** | 786.55 ± 32.97 | 827.15 ± 108.21 | 1040.46 ± 175.13 | *F*(2,3.506) = 2.733 (0.193)^B^ |
|  | **(A→C/T→G)** | 26.47 ± 11.98 | 33.29 ± 8.22 | 42.07 ± 16.4 | *F*(2,8) = 1.423 (0.296) |
|  | **(A→T/T→A)** | 48.77 ± 11.38 | 46.54 ± 22.31 | 63.42 ± 25.39 | *F*(2,8) = 0.696 (0.526) |
|  | **(C→A/G→T)** | 30.19 ± 37.79 | 40.06 ± 48.61 | 74.03 ± 56.22 | *F*(2,8) = 0.791 (0.486) |
|  | **(C→G/G→C)** | 4.99 ± 2.59 | 6.11 ± 3.12 | 7.01 ± 0.81 | *F*(2,8) = 0.564 (0.590) |
|  | **(C→T/G→A)** | 159.43 ± 28.38 (A)^A^ | 198.15 ± 21.89 (A) | 342.52 ± 36.87 (B) | *F*(2,8) = 37.419 (<0.001) |
| **Mitochondrial** | **(A→G/T→C)** | 878.46 ± 118.57 | 925.08 ± 81.82 | 922.97 ± 19.31 | *F*(2,8) = 0.338 (0.723) |
|  | **(A→C/T→G)** | 47.28 ± 16.99 | 45.98 ± 15.73 | 59.48 ± 27.22 | *F*(2,8) = 0.470 (0.641) |
|  | **(A→T/T→A)** | 34.19 ± 13.22 | 41.62 ± 12.19 | 36.16 ± 4.87 | *F*(2,8) = 0.460 (0.647) |
|  | **(C→A/G→T)** | 85.19 ± 84.32 | 89.48 ± 79.43 | 97.43 ± 8.84 | *F*(2,8) = 0.260 (0.975) |
|  | **(C→G/G→C)** | 22.76 ± 31.14 | 30.01 ± 27.41 | 26.44 ± 29.19 | *F*(2,8) = 0.061 (0.941) |
|  | **(C→T/G→A)** | 654.23 ± 787.25 | 696.13 ± 642.09 | 736.18 ± 592.63 | *F*(2,8) = 0.012 (0.988) |
| **Nuclear** | **(A→G/T→C)** | 1002.33 ± 104.22 | 930.73 ± 247.89 | 1331.09 ± 213.89 | *F*(2,8) = 3.894 (0.066) |
|  | **(A→C/T→G)** | 46.49 ± 18.90 (A) | 39.43 ± 17.54 (A) | 97.32 ± 14.88 (B) | *F*(2,8) = 10.744 (0.005) |
|  | **(A→T/T→A)** | 59.73 ± 25.29 | 46.98 ± 13.73 | 168.92 ± 69.50 | *F*(2, 3.812) = 3.997 (0.116)^B^ |
|  | **(C→A/G→T)** | 39.21 ± 14.68 | 33.28 ± 14.35 | 85.71 ± 48.98 | *F*(2,8) = 3.569 (0.078) |
|  | **(C→G/G→C)** | 19.79 ± 5.31 | 23.52 ± 12.24 | 58.29 ± 73.49 | *F*(2,8) = 1.043 (0.396) |
|  | **(C→T/G→A)** | 319.75 ± 154.41 | 300.47 ± 112.46 | 793.15 ± 499.31 | *F*(2,8) = 3.354 (0.088) |

A: Values statistically different in post-hoc tests are indicated in brackets by different letters (A or B).

B: Determined by Welch test; homogeneity of variance is not met.
